# Knowledge and attitudes toward learning disabilities among medical and nursing students in Vietnam: findings from a national cross-sectional survey

**DOI:** 10.1186/s12909-025-07956-4

**Published:** 2025-11-10

**Authors:** Quynh Hong Ngoc Nguyen, Dung Thi Thủy Nguyen, Thao Anh Hoang, Chau Anh Nguyen, Ha Ngan Nguyen, Susan De La Paz, Pranee Liamputtong, Huyen Thi Hoa Nguyen, Hieu Tran Dieu Nguyen, Phuoc Van Le

**Affiliations:** 1 College of Health Sciences, VinUniversity, Hanoi, Vietnam; 2 Department of Counseling, Higher Education, and Special Education, College of Education, University of Maryland, College Park, MD USA; 3 College of Arts and Sciences, VinUniversity, Hanoi, Vietnam; 4 Center for Environmental Intelligence, VinUniversity, Hanoi, Vietnam; 5 School of Medicine, University of California, San Francisco, CA USA

**Keywords:** Learning disabilities, Medical students, Nursing students, Vietnamese health professional education, National survey, Inclusive education

## Abstract

Learning disabilities (LD) is under-researched in medical and nursing education worldwide, especially regarding students’ perceptions of their peers with LD—an area particularly overlooked in low- and middle-income countries like Vietnam. As future healthcare professionals, whether Vietnamese medical and nursing students are aware of or accurately understand LD, and whether their perceptions may pose barriers to creating a more inclusive educational environment for peers with LD, remained unanswered. Our study investigated the knowledge and attitudes towards LD among 845 undergraduate medical and nursing students from 14 universities across Vietnam. A quantitative, cross-sectional, online questionnaire was conducted from November 2023 to February 2024, as part of a larger mixed-method exploratory study. Questions about knowledge and attitude toward LD were scored using a 4-point Likert scale. The survey results showed average knowledge and attitudes scores of 16.09/27 and 34.01/60, respectively. Pearson’s Correlation Coefficient was used to assess the relationship between knowledge and attitude scores. Associations between sociodemographic factors and these scores were analyzed using t-tests (for binary variables), one-way ANOVA (for categorical variables), and Spearman correlations (for continuous variables). Notably, students attending private universities and coming from families with higher incomes had significantly higher knowledge scores, while these factors did not influence attitude scores. The weak correlation coefficient between knowledge and attitudes suggests that comprehensive strategies are needed to meaningfully influence students’ perceptions of LD. Recommendations include integrating LD-related content into curricula, providing training for both faculty and students on LD recognition and support strategies, and establishing peer-support programs in universities throughout Vietnam. Targeted interventions should prioritize raising awareness among students from public universities and those from lower socioeconomic backgrounds.

**Supplementary Information:**

The online version contains supplementary material available at 10.1186/s12909-025-07956-4.

## Introduction

Learning disabilities (LD) refers to a diverse group of disorders characterized by significant and persistent challenges in learning and using academic skills, such as reading, writing, and/or mathematics [1]. These difficulties can persist into higher education, particularly impacting medical and nursing students due to the rigorous nature of their coursework [2]. Multiple studies have shown that medical students with LD, if detected, face significant disparities in academic performance (having lower grades, higher rate of course failure [3, 4]), mental wellbeing (experiencing fear, stigma [5]), and career prospects (concerning residency applications, professional development [3, 6]).

Despite the significant impact of LD on medical and nursing students, little has been published about LD within undergraduate medical and nursing education globally, with a particular lack of research on students’ perceptions of their peers with LD. In the Vietnamese academic context, the gap was even more pronounced as there is no specific national policy dedicated to people living with LD [7], thereby perpetuating a form of “hidden disabilities” [8, 9]. Meanwhile, a national screening study in Vietnam has estimated that 5–8% of students in primary school are facing difficulties in reading, writing, and performing mathematical skills [8], suggesting a potentially significant prevalence of LD that may persist into higher education.

Furthermore, some studies in Vietnam touched upon the misunderstanding or misclassification between LD and other conditions such as intellectual disabilities (ID), attention-deficit hyperactivity disorder (ADHD), and autism spectrum disorders (ASD); with the latter ones are sometimes viewed through highly stigmatizing lenses—such as “schizophrenia-like illnesses,” “karmic punishment,” or “family problems.” [10, 11]. Such stigmas foster shame, guilt, and social isolation for affected individuals and their families [11]. It is important to clarify that LD—referred to as Specific Learning Disorders in the DSM-5—are distinct from these conditions. According to the DSM-5, LD is not attributable to intellectual disability, sensory impairments, neurological conditions (such as pediatric stroke), adverse environmental or economic factors, inadequate instruction, or language difficulties [12]. Despite enacted inclusive education policies mandating acceptance of disabled children to public schools [13], students with these conditions are frequently rejected as being fabricated, neglected by undertrained teachers, bullied by peers, and lack access to affordable professional support [10, 11, 13]. While no specific research on LD exists in the Vietnamese context, similar patterns of stigma and inadequate support are likely to apply.

The lack of awareness and support for LD in health professional education is particularly concerning, as studies from other countries have shown that the increased academic demands of medical school can expose previously unrecognized LD, negatively affecting students’ quality of life, academic performance, and career trajectories [2]. It is important to conduct research with medical and nursing students because they are both the primary stakeholders most affected by academic pressures and support systems, and the future healthcare professionals who play a vital role in identifying, referring, and supporting individuals with LD and their families. Their knowledge and attitudes are therefore central to shaping both an inclusive educational environment and future clinical practice, ultimately influencing how LD is addressed within the broader healthcare system [14, 15]. Nevertheless, little is currently known about Vietnamese medical and nursing students’ conceptions and understanding of LD, as prior research has centered on faculty or general populations. Conducting a KAP (Knowledge, Attitude, Practice) study among this group is essential to establish baseline understanding, identify misconceptions, and uncover barriers that may hinder the development and implementation of effective, context-specific interventions [16].

Building on these gaps, our study aims to quantitatively investigate the knowledge and attitude towards LD among medical and nursing students in Vietnam. This study was also part of a larger mixed-method study, which represents, to our knowledge, the first large-scale KAP study on LD in Vietnamese health professional education. By providing insights into students’ perspectives and identifying potential misconceptions or knowledge gaps, our findings can inform evidence-based policymaking and guide the development of more inclusive educational practices in Vietnamese medical and nursing programs.

## Methods

### Study design and data collection

The mixed-method study employed a convergent parallel design, with the quantitative component being a cross-sectional online survey. Information about knowledge and attitude toward LD among Vietnamese medical and nursing students was collected during November 2023 to February 2024, using SurveyMonkey as the data collection tool. Using convenience sampling, participants were recruited through university email lists, social media, and on-campus pamphlets. This approach was selected due to time and budget constraints, as well as the absence of baseline data to guide probability sampling in this understudied population. This approach provided a timely, up-to-date snapshot of an understudied topic, and the online format ensured the broadest reach possible across Vietnamese universities.

The inclusion criteria included being undergraduate medical and nursing students from universities in Vietnam, providing written consent, and being able to complete the questionnaire in Vietnamese. In the online survey, a consent form was provided on the front page for participants to review. It clearly stated that clicking the “Proceed” button indicated they had read the form and agreed to participate voluntarily. If participants chose to withdraw during the survey, they could do so by clicking the “X” button at the top of the page, which directed them to a withdrawal form. Participants who did not complete the survey were considered to have withdrawn consent, as outlined in the consent form, and their data was excluded from the analysis. Student IDs were requested solely for verification purposes and were entirely optional. To ensure complete confidentiality, any identifiable data was immediately and permanently deleted after internal verification. No identifiable data was included in the analysis. There are 845 complete responses received from students of 14 universities across Vietnam.

### Questionnaire design

The questionnaire consisted of five sections: eligibility screening (questions no. 1–3), sociodemographic information (questions no. 4–18), knowledge about LD (question no. 19–28), attitudes towards students with LD (question no. 29–48), and university practices related to LD (questions no. 49–55). Details about the full survey questions are available in Supplementary 01. In this manuscript, we present analyses of knowledge and attitude questions; analyses of other survey components will be reported separately.

Questions about knowledge were based on diagnostic criteria of specific learning disorder (hereafter LD) in the DSM-5 [17]. The knowledge section included 9 items based on DSM-5 diagnostic criteria for LD [17], with statements covering definitions, symptoms, and contributing factors. Questions about attitudes toward LD were adapted from previously published instruments on university faculty’s perceptions of students with LD, focusing on accommodations and the legitimacy of LD-related claims. This section explored attitudes toward LD in five aspects, including: fairness and inclusivity, teaching accommodations, exam accommodations, performance expectations, and disclosure and believability [18–21].

Participant responses were recorded on a 4-point Likert scale, ranging from “0 - Strongly Disagree” and “1 - Disagree” to “2 - Agree” and “3 - Strongly Agree.”

The questionnaire was initially developed in English and then back-translated into Vietnamese to ensure accuracy. Face validity was assessed through expert reviews and piloting. Two experts independently provided feedback on content adjustments: one specializing in higher education for special education and the other with expertise in LD and other disabilities within Vietnamese higher education settings. Additionally, bilingual project members and an expert panel evaluated the questionnaire to ensure alignment with Vietnamese language, cultural norms, and comprehensive coverage of the topic.

We acknowledge that formal content validity assessment—typically involving experts rating each item—was not feasible due to limited availability of LD specialists in Vietnam. Hence, we enhanced face validity by piloting the questionnaire with 13 undergraduate medical and nursing students, to assess the readability and comprehensibity of the questionnaire, as well as problems when completing the questionnaire [16], prior to official data collection. Feedback from the pilot informed further language refinement and clarity. Students participating in the pilot were excluded from the main survey to maintain the validity of the final data. Internal reliability of the knowledge and attitudes sections was also evaluated using Cronbach’s alpha to ensure item consistency. Given the exploratory nature of our study, Cronbach’s alpha threshold of 0.7 was considered acceptable reliability [22].

### Data analysis

Descriptive statistics were computed for all sociodemographic variables, with means and standard deviations reported for continuous variables, and counts and percentages for categorical variables. Total knowledge and attitude scores were treated as continuous outcome variables by summing the points for each respective section. Reverse-coded items, including incorrect knowledge statements and negative attitudes, were scored in the opposite direction (e.g., “3 - Strongly Disagree” to “0 - Strongly Agree”) before summing. The maximum possible scores were 27 for the knowledge section and 60 for the attitudes section.

Pearson’s Correlation Coefficient was used to assess the relationship between knowledge and attitude scores. The associations between sociodemographic variables and knowledge or attitude scores was examined using bivariate analyses. Parametric tests - t-tests for binary variables and one-way ANOVA for categorical variables - were used when assumptions of normality and homogeneity of variance were met, as assessed using Shapiro-Wilk and Levene’s tests, respectively. When these assumptions were violated, non-parametric alternatives such as Spearman’s rank-order correlation were employed for continuous variables. These tests were selected for their practicality and theoretical robustness, making them well-suited for early-stage, pilot survey research, where the primary objective is to explore potential associations and refine instruments measuring knowledge and attitudes [23]. Statistically significant variables from the bivariate analysis were included in multiple linear regression models to estimate adjusted effects. Data analysis was conducted using Stata/SE 15.1 and RStudio 2022.07.2.

## Results

A total of 845 participants completed the online survey. The Cronbach’s alpha for LD knowledge was ex, indicating borderline reliability. The Cronbach’s alpha for LD attitudes was 0.760, reflecting acceptable internal consistency for this section. Further details regarding the assessment of internal reliability are provided in Supplementary 02.

Most of the participants were Medical Doctor students (88.52%) from public universities (93.73%) in Northern Vietnam (59.79%), with an average Grade Point Average (GPA) of 3.06 (± 0.38). Year 6 Medical Doctor students (30.41%) and Year 4 Bachelor of Nursing students (6.86%) were the most engaged respondents. The average age was 21.74 (± 1.86), with a higher proportion of female participants (56.21%). The majority were of the Kinh ethnicity (91.12%), while nine other ethnicities represented the remaining participants. Most participants reported no religious affiliation (90.06%).

At the time of the study, 40.12% were sharing a house with friends or partners. More than half (58.58%) reported that they grew up in families residing in rural areas during most of their first 18 years, and 39.39% had at least one parent with a full undergraduate degree. Most participants did not identify as belonging to a poor or near-poor household (93.73%).

According to the 2023 Survey on Household Living Standards by the General Statistics Office of Vietnam (GSO), households are divided into five income quintiles (each representing 20% of the population), with thresholds varying by rural or urban residence. Average monthly incomes were VND 1.22 vs. 2.26 million for Group 1 (lowest 20%), VND 2.33 vs. 3.80 million for Group 2, VND 3.52 vs. 5.03 million for Group 3, VND 4.80 vs. 7.01 million for Group 4, and VND 8.96 vs. 13.21 million for Group 5 (highest 20%), among rural and urban households, respectively [24].

These quintiles, accounting for rural and urban differences, were used to categorize the socioeconomic backgrounds in which participants grew up. The largest proportions of participants were in Group 2 (22.60%), Group 3 (27.22%), and Group 5 (26.27%), while Group 1 comprised 11.72% and Group 4 comprised 10.65%. Only 1.54% of participants did not disclose their household income. Table 1 summarizes the characteristics of study participants.

**Table 1:** Characteristics of study participants (*n* = 845)

Characteristics			Frequency	Percentage %
Study program	Bachelor of Nursing (typically 4 years)		97	11.48
	Medical Doctor (6 years)		748	88.52
Study year	Medical students	Year 1	19	2.25
		Year 2	54	6.39
		Year 3	167	19.76
		Year 4	107	12.66
		Year 5	144	17.04
		Year 6	257	30.41
	Nursing students	Year 1	3	0.36
		Year 2	7	0.83
		Year 3	19	2.25
		Year 4	58	6.86
		Year 5 (*)	10	1.18
University	By type	Public	792	93.73
		Private	53	6.27
	By region	North	501	59.29
		Middle	32	3.79
		South	312	36.92
GPA (#)	*on 4-point scale*		3.06 ± 0.38	
Gender	Female		475	56.21
	Male		347	41.07
	Non-binary/LGBT community/Others		17	02.01
	Prefer not to disclose		6	0.71
Age (^)	*in years*		21.74 ± 1.86	
Ethnicity	Kinh		770	91.12
	Dao		2	0.24
	Hoa		15	1.78
	Khmer		8	0.95
	Muong		12	1.42
	Nung		10	1.18
	Raglan		1	0.12
	San Diu		3	0.36
	Thai		3	0.36
	Tay		21	2.49
Religion	Buddhism		36	4.26
	Caodaism		1	0.12
	Catholicism		42	4.97
	Christianity		2	0.24
	Protestantism		3	0.36
	None		761	90.06
Parental education	Secondary/High school Diploma		300	35.50
	Vocational training/College degree		74	8.76
	A full undergraduate degree (Bachelor, Engineer, Medical Doctor, or equivalent)		332	39.29
	A full graduate degree (Master and/or PhD, Specialized Professional Degree)		120	14.20
	Do not know		19	2.25
Living status	Living with parents		149	17.63
	Sharing house with friends/partners		339	40.12
	Living with relatives/extended family		112	13.25
	Living alone		245	28.99
Living area	Rural		495	58.58
	Urban		350	41.42
Household	Poor		13	1.54
	Near poor		40	4.73
	Average		792	93.73
Income group per person per month in each household (classified by Vietnam GSO, accounted for living area as rural or urban)	Group 1		99	11.72
	Group 2		191	22.60
	Group 3		230	27.22
	Group 4		90	10.65
	Group 5		222	26.27
	Not disclosing		13	1.54
TOTAL			845	100.00

(*): Advanced Nursing Program at one university in Hanoi (4.5 years)

(#): Value reported in Mean ± SD; Note: Missing value = 354/845 = 41.89% missing value, due to not willing to disclose GPA

(^): Value reported in Mean ± SD

### Knowledge scores, attitude scores and their correlation

**Fig. 1:**
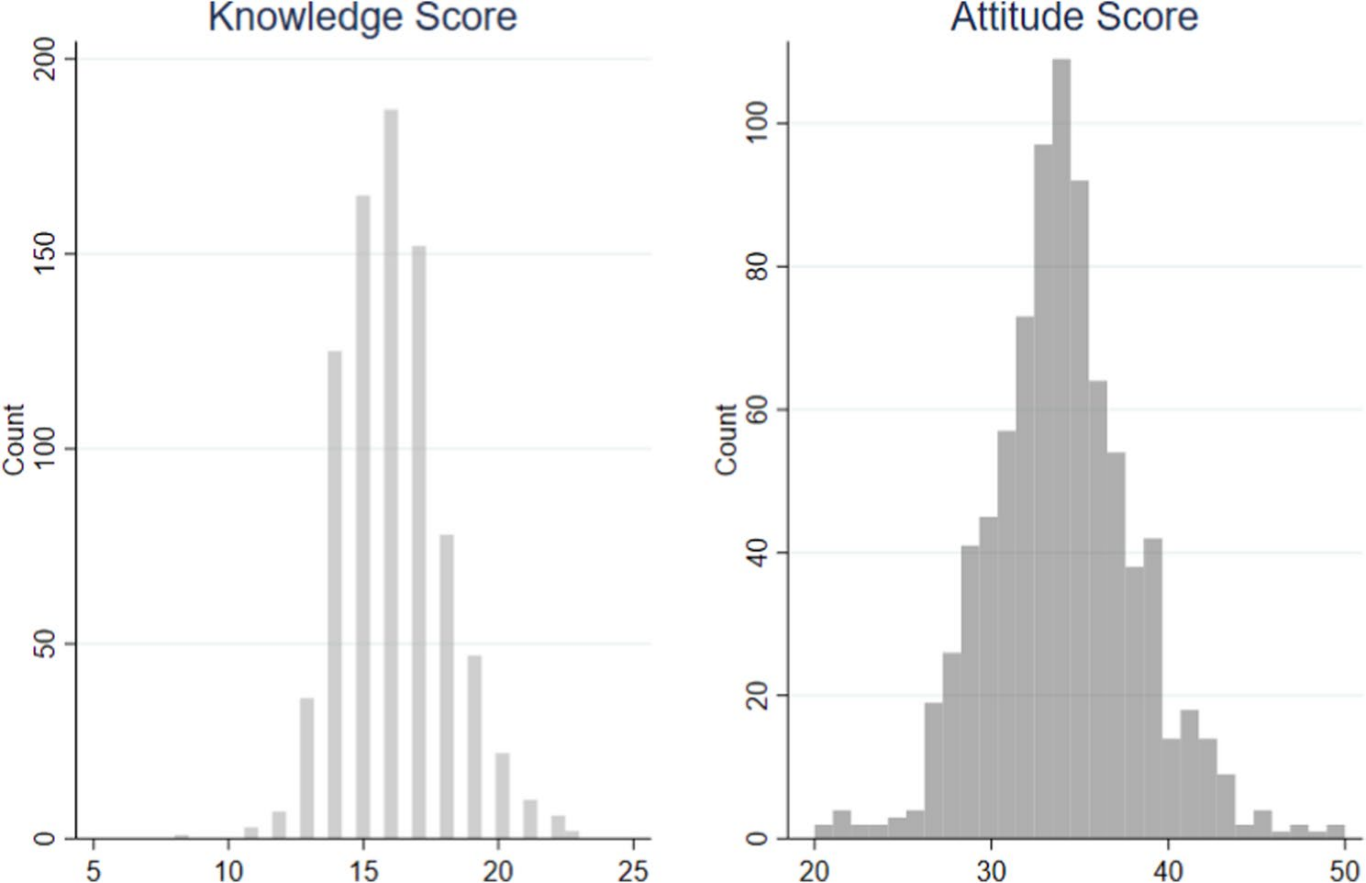
Histograms of Knowledge and Attitude Scores (*n* = 845)

**Fig. 2:**
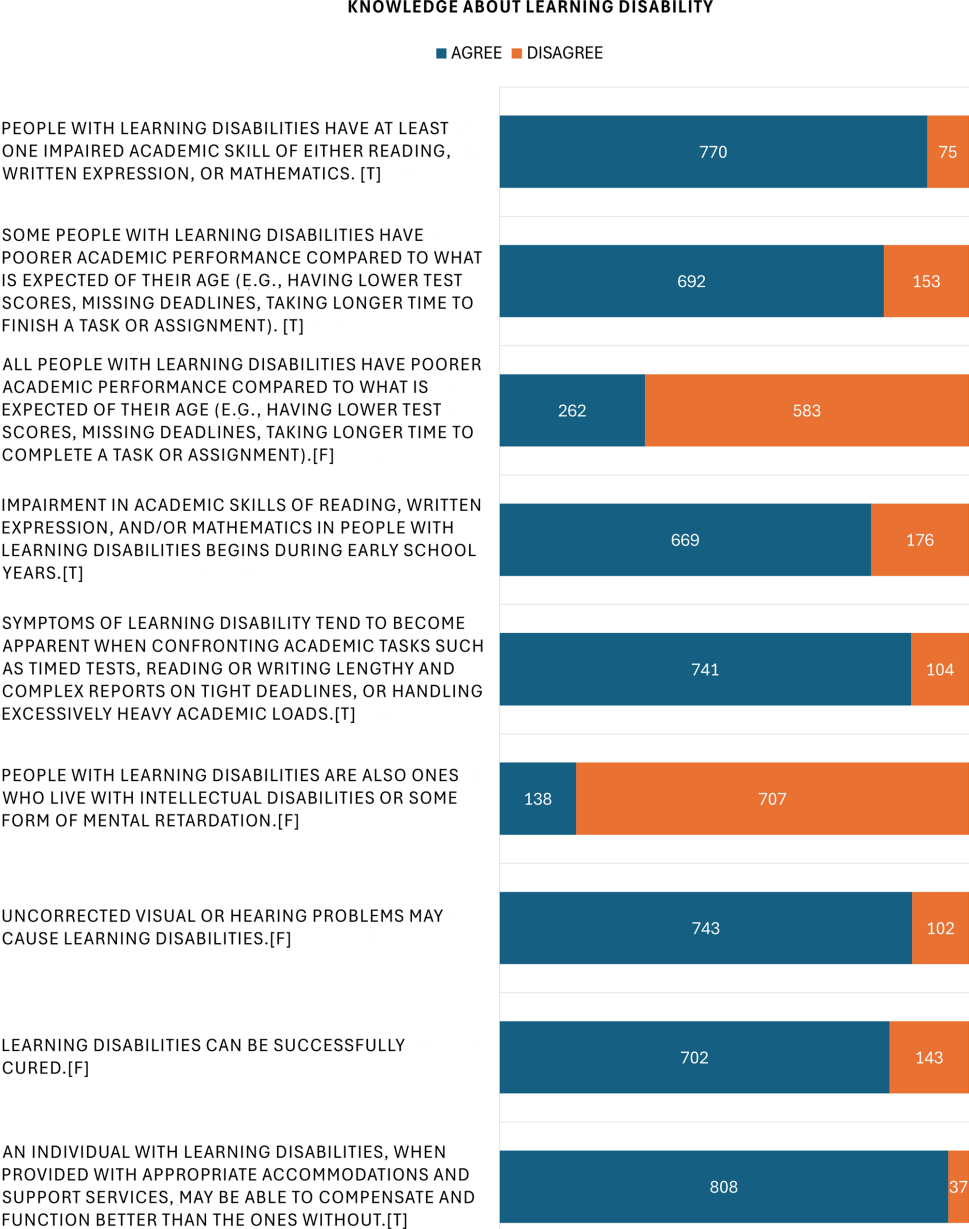
Participants’ Knowledge about LD (*n* = 845); Abbreviation: [T], True; [F], False

Table 2 shows that the knowledge scores had a weak but statistically significant positive correlation with attitude score (*r* = 0.116, *p* < 0.001). Figure 1 summarizes the distributions of knowledge and attitude scores. On a scale of 0–27, the average knowledge score was 16.09 ± 1.91. Participants generally answered correctly on most aspects of LD, including definition, symptoms, aggravating factors, and prognosis. However, a common misconception was that “Learning disabilities can be successfully cured,” which most participants believed to be true (Fig. 2). On a scale of 0–60, the average attitude score was 34.01 ± 4.7. Under the theme of fairness and inclusivity, most participants agreed that universities should implement special considerations to support students with learning disabilities (LD), and viewed such measures as fair to all students. Regarding performance expectations, the majority expressed confidence in the academic abilities of students with LD, believing they are capable of completing university programs and competing academically with their peers. Participants generally expressed positive attitudes towards teaching and exam accommodations for students with LD.

**Table 2:** Descriptive statistics and Pearson’s correlation coefficient between knowledge and attitude scores (*n* = 845)

Measurement	Descriptives							Pearson’s Correlation Test	
	Mean	Standard Deviation	Min	Max	25% percentile	75% percentile	90% percentile	Correlation Coefficient	*p*-value
Knowledge Score	16.09	1.91	8	23	15	17	19	0.116	< 0.001
Attitude Score	34.01	4.17	16	51	32	36	39

**Fig. 3:**
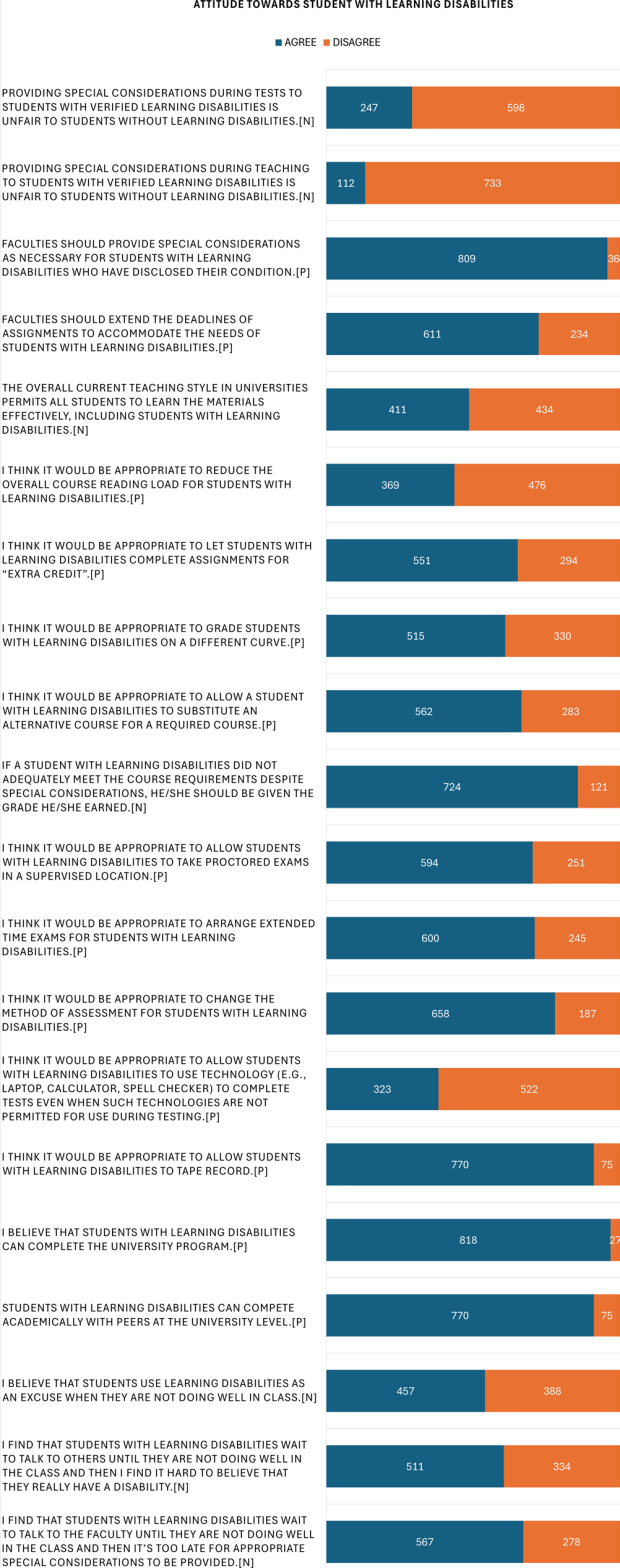
Participants’ Attitude about LD (*n* = 845); Abbreviation: [P], Positive; [N], Negative

However, certain accommodations received Less support. Students were less favorable towards reducing course reading loads, adjusting grades for students with LD who did not meet requirements despite special considerations, and permitting the use of technology during tests when normally prohibited. Attitudes were also more negative in the theme of disclosure and believability of LD. Specifically, 457 (54.08%) believed that other students might use LD as an excuse for poor performance, 511 (60.47%) doubted the Legitimacy of LD due to delayed disclosure, and 567 (67.1%) felt that timely special considerations were hindered by such delays (Fig. 3).

### Relationships between university type, household poverty, income group and knowledge score

Bivariate analysis revealed significant associations between knowledge score and university type (*p* = 0.003), household poverty (*p* = 0.010), and income group (*p* = 0.044) (Table 3). Tukey’s post-hoc test found a significant difference in knowledge scores between participants from average and poor household (*p* = 0.042), while no significant differences were found between pairs of income groups (Table 4).

**Table 3:** Bivariate and multivariate analysis for knowledge score (*n* = 845)

Independent Variables		Knowledge Score		Bivariate Test		Multiple Linear Regression Model		
		Mean ± SD	Freq.	t (T-test)/ F (ANOVA)/*r* (Spearman)	*p*-value	Coefficient	95% CI	*p*-value
Study program	Medicine	16.08 (1.93)	748	−0.367	0.714	ns	ns	ns
	Nursing	16.15 (1.80)	97					
University Type	Public	16.04 (1.89)	792	−3.006	0.003*	-	-	-
	Private	16.85 (2.06)	53			0.712	(0.162–1.262)	0.011*
University region	North	16.14 (1.89)	501	0.64	0.529	ns	ns	ns
	Middle	16.16 (1.85)	32					
	South	15.99 (1.96)	312					
Gender	Others	16.09 (2.02)	23	0.8	0.448	ns	ns	ns
	Male	15.99 (2.06)	347					
	Female	16.16 (1.80)	475					
Age				−0.001	0.978	ns	ns	ns
Ethnic	Kinh	16.10 (1.93)	770	0.668	0.505	ns	ns	ns
	Other minorities	15.95 (1.73)	75					
Religion	Non-religious	16.09 (1.92)	761	0.322	0.748	ns	ns	ns
	Religious	16.02 (1.88)	84					
GPA	Not willing to disclose GPA	16.01 (0.10)	354	−0.98	0.326	ns	ns	ns
	Disclosed GPA	16.14 (0.09)	491					
Study year (MD)	1	16.89 (1.73)	19	1.07	0.374	ns	ns	ns
	2	16.13 (1.78)	54					
	3	15.94 (1.99)	167					
	4	16.01 (1.89)	107					
	5	15.99 (1.73)	144					
	6	16.18 (2.05)	257					
Study year (BN)	1	14.33 (0.58)	3	1.2	0.317	ns	ns	ns
	2	16.57 (2.07)	7					
	3	15.89 (1.94)	19					
	4	16.19 (1.67)	58					
	5	16.7 (2.21)	10					
Living status	Living with parents	16.17 (1.95)	149	2.57	0.053	ns	ns	ns
	Sharing house with friends/partners	16.18 (1.97)	339					
	Living with relatives/extended family	16.29 (2.07)	112					
	Living alone	15.81 (1.72)	245					
Parental education (*)	Secondary/High school Diploma	16.01 (1.78)	300	0.61	0.607	ns	ns	ns
	Vocational training/College degree	16.30 (1.73)	74					
	A full undergraduate degree (Bachelor, Engineer, Medical Doctor, or equivalent)	16.14 (2.12)	332					
	A full graduate degree (Master and/or PhD, Specialized Professional Degree)	16.19 (1.76)	120					
Household	Poor	14.85 (3.29)	13	4.60	0.0103*	-	-	-
	Near poor	15.55 (1.54)	40			1.559	(0.313–2.806)	0.014*
	Average	16.14 (1.89)	792			2.003	(0.882–3.125)	< 0.001*
Living area	Rural	15.99(1.83)	495	−1.767	0.078	ns	ns	ns
	Urban	16.23 (2.02)	350					
Income group (^)	Group 1	15.71 (1.78)	99	2.46	0.044*	-	-	-
	Group 2	15.88 (1.96)	191			0.091	(−0.367–0.549)	0.696
	Group 3	16.14 (1.89)	230			0.304	(−0.145–0.753)	0.185
	Group 4	16.44 (1.82)	90			0.562	(0.017–1.107)	0.043*
	Group 5	16.16 (1.92)	222			0.226	(−0.231–0.683)	0.332

*Abbreviation*: *ns* not significant, *MD* Medical Doctor, *BN* Bachelor of Nursing, *CI* Confidence interval

(#) Responses indicating “Do not know” for parental education are excluded from analysis

(^) Responses with missing household income information are excluded from analysis

* *p* ≤ 0.05

**Table 4:** Post hoc Tukey HSD (Honestly significant Difference) test results for ANOVA: Between-Group differences in knowledge and attitude scores (*n* = 845)

Measurement	Variable	Group	Group	Mean Difference	Standard Error	95% CI	*p*-value
Knowledge Score	Household	Poor	Near poor	1.383	0.621	(−0.074 −2.841)	0.067
			Average	1.964	0.549	(0.676–3.252)	0.001*
		Near poor	Average	0.581	0.306	(−0.137–1.298)	0.140
	Income group	Group 1	Group 2	0.191	0.234	(−0.449–0.830)	0.926
			Group 3	0.446	0.227	(−0.175–1.067)	0.286
			Group 4	0.733	0.276	(−0.021–1.487)	0.061
			Group 5	0.437	0.228	(−0.187–1.062)	0.310
		Group 2	Group 3	0.255	0.184	(−0.249–0.759)	0.640
			Group 4	0.542	0.242	(−0.118–1.203)	0.165
			Group 5	0.246	0.186	(−0.262–0.755)	0.676
		Group 3	Group 4	0.287	0.235	(−0.356–0.930)	0.739
			Group 5	−0.009	0.178	(−0.494–0.477)	1.000
		Group 4	Group 5	−0.296	0.236	(−0.942–0.350)	0.721
Attitude Score	Income group	Poor	Near poor	−3.783	1.327	(−6.900 - −0.667)	0.012*
			Average	−2.525	1.173	(−5.279 −0.229)	0.080
		Near poor	Average	1.258	0.654	(−0.276–2.793)	0.132

*Abbreviation*: *CI* Confidence interval

* *p* ≤ 0.05

Multiple linear regression showed that private university students had higher knowledge scores than public university students by 0.71 (95% CI: 0.16,1.26; *p* = 0.011). Students from near poor and average household scored 1.55 (95% CI: 0.31, 2.81; *p* = 0.014) and 2.00 (95% CI: 0.88, 3.12; *p* < 0.001) points higher than those from poor household. Higher income groups were also associated with better knowledge scores (Table 3).

### Relationships between household poverty and attitude score

Household poverty was the sole predictor of attitude scores (*p* = 0.026), with Tukey’s post-hoc test identifying a significant difference between participants from poor and near-poor household (*p* = 0.023) (Tables 4 and 5 ). Multiple linear regression showed that, compared to those coming from poor households, students growing up in near poor and average households had Less positive attitude scores by 3.78 points (95% CI: −6.39, −1.18; *p* = 0.004) and 2.53 points (95% CI: −4.83, −0.22; *p* = 0.032), respectively (Table 5).

**Table 5:** Bivariate and multivariate analysis for attitude score (*n* = 845)

Independent Variables		Attitude Score		Bivariate Test		Multiple Linear Regression Model		
		Mean ± SD	Freq.	t (T-test)/ F (ANOVA)/*r* (Spearman)	*p*-value	Coefficient	95% CI	*p*-value
Study program	Medicine	34.02 (4.19)	748	0.259	0.796	ns	ns	ns
	Nursing	33.91 (4.08)	97					
University Type	Public	34.02 (4.16)	792	0.223	0.824	ns	ns	ns
	Private	33.89 (4.42)	53			0.712	(0.162–1.262)	0.011*
University region	North	34.06 (4.05)	501	0.53	0.591	ns	ns	ns
	Middle	33.28 (4.55)	32					
	South	34.00 (4.33)	312					
Gender	Others	32.57 (3.64)	23	2.3	0.101	ns	ns	ns
	Male	34.28 (4.41)	347					
	Female	33.89 (4.01)	475					
Age				0.02	0.537	ns	ns	ns
Ethnic	Kinh	33.99 (4.19)	770	−0.527	0.598	ns	ns	ns
	Other minorities	34.25 (4.05)	75					
Religion	Non-religious	34.04 (4.18)	761	0.575	0.565	ns	ns	ns
	Religious	33.76 (4.15)	84					
GPA	Not willing to disclose GPA	33.80 (4.23)	354	−1.23	0.218	ns	ns	ns
	Disclosed GPA	34.16 (4.13)	491					
Study year (MD)	1	34.37 (3.74)	19	2.22	0.051	ns	ns	ns
	2	35.46 (3.72)	54					
	3	33.76 (4.51)	167					
	4	33.54 (4.41)	107					
	5	33.62 (3.76)	144					
	6	34.30 (4.18)	257					
Study year (BN)	1	33 (2)	3	0.43	0.788	ns	ns	ns
	2	35.14 (6.31)	7					
	3	34.26 (4.64)	19					
	4	33.55 (3.73)	58					
	5	34.7 (3.92)	10					
Living status	Living with parents	34.24 (4.99)	149	1.93	0.124	ns	ns	ns
	Sharing house with friends/partners	33.92 (4.08)	339					
	Living with relatives/extended family	34.74 (4.45)	112					
	Living alone	33.66 (3.57)	245					
Parental education (*)	Secondary/High school Diploma	34.08 (4.00)	300	0.17	0.914	ns	ns	ns
	Vocational training/College degree	33.91 (4.92)	74					
	A full undergraduate degree (Bachelor, Engineer, Medical Doctor, or equivalent)	33.88 (4.14)	332					
	A full graduate degree (Master and/or PhD, Specialized Professional Degree)	34.11 (4.17)	120					
Household	Poor	36.31 (4.57)	13	3.69	0.026*	-	-	-
	Near poor	32.8 (3.98)	40			−3.783	(−6.389 - −1.178)	0.004*
	Average	34.03 (4.16)	792			−2.525	(−4.827 - −0.223)	0.032*
Living area	Rural	34.13 (3.86)	495	0.999	0.318	ns	ns	ns
	Urban	33.84 (4.58)	350					
Income group (^)	Group 1	34.16 (4.35)	99	1.04	0.387	ns	ns	ns
	Group 2	34.30 (4.04)	191			0.091	(−0.367–0.549)	0.696
	Group 3	33.8 (4.08)	230			0.304	(−0.145–0.753)	0.185
	Group 4	33.31 (4.20)	90			0.562	(0.017–1.107)	0.043*
	Group 5	34.04 (4.23)	222			0.226	(−0.231–0.683)	0.332

*Abbreviation*: *ns* not significant, *MD* Medical Doctor, *BN* Bachelor of Nursing, *CI* Confidence interval

(#) Responses indicating “Do not know” for parental education are excluded from analysis

(^) Responses with missing household income information are excluded from analysis

* *p* ≤ 0.05

## Discussion

Our study demonstrates that knowledge and attitudes toward LD among Vietnamese medical and nursing students are low, with mean knowledge and attitude scores of 16.09 out of 27 (59.6%) and 34.01 out of 60 (56.7%), respectively. This indicates a need for improvement in both awareness and inclusivity. Notably, students from higher-income families and private universities tended to have higher knowledge scores, suggesting that socioeconomic and institutional factors play a role in shaping understanding of LD. However, these advantages in knowledge do not necessarily translate to more positive attitudes; in fact, we observed that students from lower-income backgrounds reported more accepting attitudes towards peers with LD. This may reflect greater empathy among disadvantaged students or possibly a different socialization process. The weak correlation between knowledge and attitudes suggests that interventions must address both areas, rather than relying on education alone to shift perceptions.

Our findings are consistent with previous research showing low levels of knowledge and tolerance of individuals with LD across various so-called stakeholder groups, including LD and non-LD university students [25, 26], teaching faculty [27], community workers [18, 28], general practitioners [29], and the general public [30]. Comparative Israeli studies in settings with greater LD awareness reported higher knowledge and attitude scores among university students [20, 31], underscoring the gap in Vietnamese health education. Notably, at least one of these studies [20] used the attitude questionnaire adapted from the same source authors as ours [19, 32], which reinforces the comparison that Vietnamese students lag in their knowledge and positive attitudes towards people living with LD.

The association between higher socioeconomic status (SES) and greater LD knowledge in our findings aligns with existing literatures. It appeared that less well-educated people from lower economic background, even those who fulfilled the lay definition of dyslexia being ‘discrepancy between IQ and reading and spelling skills’, are less likely to self-report as dyslexic, partially owing to them being unaware of such definition [33]. In the context of ID, higher socioeconomic status (SES) is also linked to greater mental health literacy [34], while children with ID from economically disadvantaged backgrounds often face barriers to essential resources and care [35]. A similar explanation applies to our findings: students from families with higher SES have the financial means and cultural guidance towards a broader range of educational resources, including those related to LD. As a result, interventions aimed at improving LD knowledge should particularly focus on students from lower SES backgrounds, as they may benefit most from institutional support.

Interestingly, students from lower SES backgrounds expressed more positive attitudes toward peers with LD, a finding that diverges from trends observed in high-income countries. While prior literature suggests that higher SES correlates with greater disability awareness and tolerance, our data appear to reveal a contextually responsive in the Vietnamese setting [36]. Rather than viewing this as a contradiction, it may reflect a distinct sociocultural mechanism, students from disadvantaged backgrounds may possess heightened empathy toward peers facing academic and social challenges, deriving from their own lived experiences of marginalization, limited access to educational resources, and systemic barriers [36, 37]. While this sense of shared struggle may be less apparent in high-income countries with robust welfare systems – the setting of most existing LD research, it could be more pronounced in Vietnam, where limited social support may foster greater identification with peers facing similar hardships. This may help explain why students from poorer households in our study expressed more positive attitudes toward peers with LD than their near-poor or higher-income counterparts.

Attending a private university was also associated with better LD knowledge among our participants. However, this should be interpreted cautiously, as many of the private university students in our study were enrolled in English-exclusive curricula. Given that LD resources are more widely available in English, rather than in Vietnamese, these students may have had an advantage over their peers simply due to their English proficiency.

Moreover, the weak correlation between knowledge and attitude scores in our results, which also mirrored in other studies [27, 29]^38^– [40] suggests that knowledge-based interventions alone may not be enough to improve attitudes towards students with LD. Research consistently shows that one of the most effective factors in fostering positive attitudes is having direct contact with individuals with LD [21, 30]^41^– [44]. Therefore, combining awareness-raising programs with inclusive environments where students with and without LD interact may be necessary to combat misconceptions and reduce negative attitudes.

Many participants were generally supportive of accommodations for students with LD, yet skepticism about the legitimacy of LD diagnoses and the timing of disclosure persisted. As a “hidden” disability, the absence of an official diagnostic test in Vietnam severely disadvantages students with LD, leaving them unable to prove the validity of their condition. Establishing Ministry of Health-endorsed diagnostic criteria for LD is essential to address this skepticism, as recommended by the National Joint Committee on Learning Disability (NJCLD) [45]. However, even when diagnosed, many students with LD still hesitate to disclose their condition due to fear of discrimination [26], anxiety [46], or concerns about being perceived as exploiting the system [47]. Overcoming these misconceptions will require a dual approach: educating students without LD on the psychological barriers faced by their peers, while encouraging students with LD to seek help early. To facilitate these changes, the universities play a role in educating students and faculties about LD, devising suitable accommodations, and creating policy to support the students with LD.

Unlike students at the K-12 level, students with disabilities in higher education in western countries (including the United States) must self-identify as having disabilities to a designated campus unit (i.e., Disability Services Office) to request accommodations [48]. In addition, students must provide documentation about the nature of their disability and the services they received before starting college. In Vietnam, the assessment of dyslexia varies, depending on whether families want to seek a disability certificate, or to inform intervention and educational support, or for research purposes [49]. Reading rate, the ability to read nonsense words, number of written words generated, and type of spelling errors have been recommended as ways to identify children with dyslexia in Vietnam [49], but clearly more work is needed to validate these methods. While no comparable methods are yet available in higher education, it seems reasonable that future research in this area focuses on developing ways to screen for core deficits such as phonological processing problems (including verbal working memory and speeded sentence-level reading) [50] and rapid naming [51] as these are hallmark underlying characteristics of one type of LD.

International evidence further underscores the significance of providing information about LD in health professional education. For example, in one study conducted in the United States, LD accounted for 21.5% of all declared disabilities among medical students [46]. Similarly, in the United Kingdom, LDs are the most commonly declared disability among medical students, with declarations increasing from 1.4% (among 2002 entrants) to 4.6% (among 2018 entrants) over the past two decades [47]. Regionally, mention of interventions for LD is limited in Asian countries, with notable exceptions in Cambodia and Malaysia. In Cambodia, integrated classes exist for students who are blind, deaf, or have LD, but remote areas often lack these special needs schools and inclusive mainstream schools [52]. In Malaysia, besides special education schools, integrated program, inclusive education programs, there are also rehabilitation and early learning program available [53]. These initiatives show that inclusive education was underway in the Asian region, which could help increase exposure of students with LD to foster positive attitudes.

By presenting context-specific data from Vietnam, our study addresses this global evidence gap and provides a foundation for policy and curriculum development aimed at disability inclusion in health professions education, both nationally and across the broader region. Our findings also contributes to the objectives of Sustainable Development Goal 4 (SDG 4) for equitable education and lifelong learning opportunities for all [54], as well as regional strategies such as the ASEAN “Masterplan 2025: Mainstreaming the Rights of Persons with Disabilities,” which currently lacks a specific emphasis on LD in most member countries [52]. By generating context-specific data, this research informs efforts to enhance inclusivity within educational settings and supports the development of disability-inclusive practices in medical and nursing education, both nationally and internationally.

### Limitations

This study has several limitations. The survey items were adapted from international studies, which may not fully capture the unique aspects of the Vietnamese academic context. While we assessed face validity through independent review by two experts, we were unable to conduct a formal content validity assessment—such as having multiple LD specialists systematically rate each item to calculate a content validity index [16]—due to the limited availability of LD experts in the Vietnamese educational context. Nonetheless, we sought to maximize validity by incorporating expert feedback and student pilot testing to refine the questionnaire [16] prior to official data.

Furthermore, as the data were self-reported, participants may have had the opportunity to look up information about LD while completing the questionnaire, potentially resulting in inflated knowledge scores. Social desirability (SD) bias is another potential limitation in self-reported, online surveys, as participants may be inclined to provide socially acceptable responses rather than their genuine views [55, 56], and by disabling skipping or backtracking within the survey [57]. Additionally, confidentiality assurances were provided during the consent process, which may have encouraged honesty but could also have inadvertently increased suspicion and reinforced bias [58].

Additionally, the use of convenience sampling may introduce selection bias; students who were more interested in, or familiar with, LD may have been more likely to participate, potentially affecting the generalizability of the findings. To address this limitation, we aimed to recruit as large and diverse a sample as possible, resulting in 845 participants from 14 universities across multiple geographic regions and various years of study in both medical and nursing programs. This approach aligns with best practices for convenience sampling, which recommend recruiting “as many participants or cases as possible” to enhance representativeness [59]. The commonly cited rule of thumb for such studies is 10–15 observations per parameter [60], which our sample size meets. Moreover, because no prior KAP studies on LD exist in Vietnamese health professional education, there were no effect size estimates available for power calculations. This absence of baseline data further supports our use of the convenience sampling approach. While caution should be exercised in generalizing the results, the large and heterogeneous sample provides reasonable confidence in the stability and relevance of the findings.

Lastly, the Cronbach’s alpha for the LD knowledge scale was slightly below the commonly accepted threshold of 0.70. However, this could still be considered reasonable given the exploratory nature of the study, the absence of widely validated instruments specific to learning disability knowledge, and the known tendency of Cronbach’s alpha to underestimate internal consistency in scales with a small number of items (nine in this case) [61]. Future research should aim to improve the scale’s reliability through factor analysis and item-level psychometric evaluation.

## Conclusions

This study offers the first quantitative assessment of knowledge and attitudes toward LD among medical and nursing students in Vietnam. The moderate scores in knowledge and attitudes reflect a foundational understanding of LD, influenced by factors such as university type, household poverty, and background family income. Notably, the observed gap between knowledge and attitude scores highlights that disseminating knowledge alone is insufficient to shift perceptions of LD. This underscores the need for multifaceted approaches that address not only knowledge but also attitudes and awareness to foster a more LD-inclusive learning environment.

Our findings have significant implications for policy and practice in Vietnam. By providing quantifiable data on students’ perceptions of LD, this study offers a foundation for developing evidence-based, context-specific educational reforms in Vietnamese medical education. Future research could include individual item analysis to refine and validate the questionnaires, contributing to a more robust framework for assessing medical students’ knowledge and attitudes toward LD. Such efforts are essential for creating inclusive and supportive learning environments, ultimately enhancing the quality of medical education and healthcare delivery related to LD in Vietnam.

## Supplementary Information

Supplementary Material 1.

Supplementary Material 2.

## Data Availability

The datasets used and/or analysed during the current study are available from the corresponding author on reasonable requests.

## Abbreviations

LD: Learning disabilities
ADHD: Attention deficit hyperactivity disorder
ASD: Autistic spectrum disorders
KAP: Knowledge - Attitude - Practice
DSM-5: The Diagnostic and Statistical Manual of Mental Disorders 5^th^ Edition (DSM-5)
GSO: The General Statistics Office of Vietnam
HSD: Honestly Significant Difference
ID: Intellectual disabilities
SES: Socioeconomic status
GPA: Grade Point Average
