# Supplementary material for: Knowledge and attitudes toward learning disabilities among medical and nursing students in Vietnam: findings from a national cross-sectional survey
Source: BMC Med Educ. 2025 Nov 10;25:1567. doi: 10.1186/s12909-025-07956-4 (PMC12604178; doi:10.1186/s12909-025-07956-4)
Supplement: Supplementary file 1 — Supplementary Material 1. [file 12909_2025_7956_MOESM1_ESM.docx]

| **Internal Reliability for LD Knowledge** | | | | |
| --- | --- | --- | --- | --- |
| **Item** | **Item-Test Correlation** | **Item-Rest Correlation** | **Average Interim Covariance** | **Cronbach's Alpha** |
| People with learning disabilities have at least one impaired academic skill of either reading, written expression, or mathematics. | 0.547 | 0.388 | 0.068 | 0.645 |
| **Some** people with learning disabilities have poorer academic performance compared to what is expected of their age (e.g., having lower test scores, missing deadlines, taking longer time to finish a task or assignment) | 0.617 | 0.459 | 0.063 | 0.629 |
| **All** people with learning disabilities have poorer academic performance compared to what is expected of their age (e.g., having lower test scores, missing deadlines, taking longer time to complete a task or assignment) | 0.638 | 0.443 | 0.059 | 0.631 |
| Impairment in academic skills of reading, written expression, and/or mathematics in people with learning disabilities begins during early school years | 0.569 | 0.408 | 0.066 | 0.641 |
| Symptoms of learning disability tend to become apparent when confronting academic tasks such as timed tests, reading or writing lengthy and complex reports on tight deadlines, or handling excessively heavy academic loads. | 0.614 | 0.469 | 0.064 | 0.629 |
| People with learning disabilities are also ones who live with intellectual disabilities or some form of mental retardation. | 0.484 | 0.27 | 0.071 | 0.672 |
| Uncorrected visual or hearing problems may cause learning disabilities. | 0.443 | 0.266 | 0.074 | 0.669 |
| Learning disabilities can be successfully cured. | 0.405 | 0.232 | 0.076 | 0.675 |
| An individual with learning disabilities, when provided with appropriate accommodations and support services, may be able to compensate and function better than the ones without. | 0.439 | 0.257 | 0.074 | 0.671 |
| **Overall** |  |  | 0.068 | **0.68** |

| **Internal Reliability for LD Attitude Score** | | | | |
| --- | --- | --- | --- | --- |
| **Item** | **Item-Test Correlation** | **Item-Rest Correlation** | **Average Interim Covariance** | **Cronbach's Alpha** |
| Providing special considerations during tests to students with verified learning disabilities is unfair to students without learning disabilities. | 0.364 | 0.247 | 0.054 | 0.756 |
| Providing special considerations during teaching to students with verified learning disabilities is unfair to students without learning disabilities. | 0.393 | 0.284 | 0.054 | 0.753 |
| Faculties should provide special considerations as necessary for students with learning disabilities who have disclosed their condition. | 0.327 | 0.237 | 0.056 | 0.756 |
| Faculties should extend the deadlines of assignments to accommodate the needs of students with learning disabilities. | 0.521 | 0.429 | 0.052 | 0.743 |
| The overall current teaching style in universities permits all students to learn the materials effectively, including students with learning disabilities. | 0.372 | 0.245 | 0.054 | 0.757 |
| I think it would be appropriate to reduce the overall course reading load for students with learning disabilities. | 0.499 | 0.398 | 0.052 | 0.745 |
| I think it would be appropriate to let students with learning disabilities complete assignments for “extra credit”. | 0.513 | 0.416 | 0.052 | 0.744 |
| I think it would be appropriate to grade students with learning disabilities on a different curve. | 0.468 | 0.362 | 0.052 | 0.748 |
| I think it would be appropriate to allow a student with learning disabilities to substitute an alternative course for a required course. | 0.497 | 0.4 | 0.052 | 0.745 |
| If a student with learning disabilities did not adequately meet the course requirements despite special considerations, he/she should be given the grade he/she earned. | 0.342 | 0.244 | 0.055 | 0.756 |
| I think it would be appropriate to allow students with learning disabilities to take proctored exams in a supervised location. | 0.466 | 0.368 | 0.053 | 0.747 |
| I think it would be appropriate to arrange extended time exams for students with learning disabilities. | 0.555 | 0.472 | 0.051 | 0.74 |
| I think it would be appropriate to change the method of assessment for students with learning disabilities. | 0.517 | 0.429 | 0.052 | 0.743 |
| I think it would be appropriate to allow students with learning disabilities to use technology (e.g., laptop, calculator, spell checker) to complete tests even when such technologies are not permitted for use during testing. | 0.475 | 0.364 | 0.052 | 0.747 |
| I think it would be appropriate to allow students with learning disabilities to tape record. | 0.399 | 0.314 | 0.055 | 0.752 |
| I believe that students with learning disabilities can complete the university program. | 0.209 | 0.119 | 0.058 | 0.762 |
| Students with learning disabilities  can compete academically with peers at the university level. | 0.261 | 0.158 | 0.057 | 0.761 |
| I believe that students use learning disabilities as an excuse when they are not doing well in class. | 0.404 | 0.277 | 0.053 | 0.755 |
| I find that students with learning disabilities wait to talk to **others** until they are not doing well in the class and then I find it hard to believe that they really have a disability. | 0.431 | 0.315 | 0.053 | 0.751 |
| I find that students with learning disabilities wait to talk to the **faculty** until they are not doing well in the class and then it’s too late for appropriate special considerations to be provided. | 0.452 | 0.343 | 0.053 | 0.749 |
| **Overall** |  |  | 0.053 | **0.76** |
