# Supplementary material for: Knowledge and attitudes toward learning disabilities among medical and nursing students in Vietnam: findings from a national cross-sectional survey
Source: BMC Med Educ. 2025 Nov 10;25:1567. doi: 10.1186/s12909-025-07956-4 (PMC12604178; doi:10.1186/s12909-025-07956-4)
Supplement: Supplementary file 2 — Supplementary Material 2. [file 12909_2025_7956_MOESM2_ESM.docx]

**QUESTIONNAIRE: Learning Disabilities among Medical and Nursing Students in Vietnam:  A Knowledge, Attitude, and Practice (KAP) Study**

1. Demographic

|  | **No.** | **Questions** | **Answers** |
| --- | --- | --- | --- |
| ***Screening Question*** | ***1*** | Are you currently in training to become a Medical Doctor or a Bachelor of Nursing? | 1. Medical Doctor (6 years) 2. Bachelor of Nursing (4 years)  3. Others  *If OTHERS => Thank you for answer; however, you do not belong to our study’s target population. Thank you for your interest.* |
| ***Screening Question*** | ***2*** | Are you a medical student/ nursing student at the following universities? | 1. Hanoi Medical University  2. Vietnam National University – Hanoi  3. VinUniversity  4. Phenikaa University  5. University of Medicine and Pharmacy at Ho Chi Minh city  6. Pham Ngoc Thach University of Medicine  7. Vietnam National University – Ho Chi Minh city  8. Hong Bang International University  9. Hue University of Medicine and Pharmacy  10. Hai Phong University of Medicine and Pharmacy  11. Thai Binh University of Medicine and Pharmacy  12. Thai Nguyen University Of Medicine and Pharmacy  13. Can Tho University of Medicine and Pharmacy  14. Vietnam Military Medical University 00. None of the above |
| ***Screening Question*** | **3** | Please verify yourself by providing your student ID card.  *This is optional and for verification purpose only.* | *OPTIONAL* |
|  | **4** | What is your gender? | 1. Male 2. Female 3. Non-binary/LGBT community/ Others 4. Preferred not disclosing |
|  | **5** | What year were you born? | *Fill out birth year* |
|  | **6** | What is your nationality? | 1: Vietnamese 2: Others, please specify |
|  | **7** | What is your ethnicity? | 1. Kinh 2. Tày 3. Thái 4. Mường 5. Khơ Me 6. Others, please specify |
|  | **8** | What is your religion? | 1. None 2. Buddism 3. Christianity 4. Catholicism 5. Protestantism 6. Other, please specify |
|  | **9** | What year of training are you in currently? | 1. First year  2. Second year  3. Third year  4. Fourth year  5. Fifth year  6. Sixth year |
|  | **10** | What is your current cumulative GPA at your university (on scale of 0-10)? | *Fill out current cumulative GPA* **Do not know/ Do not want to disclose, please leave blank* |
|  | **11** | What is your current cumulative GPA at your university (on scale of 0-4)? | *Fill out current cumulative GPA* **Do not know/ Do not want to disclose, please leave blank* |
|  | **12** | What is your **current** living situation (for most of the time, who do you live with)? | 1. Living with parents 2. Sharing house with friends/ partners 3. Living with relatives/ extended family 4. Living alone 5. Other, please specify |
|  | **13** | Which of the following best describes the educational experience of your parent/guardian (the one that has highest educational experience in your family)? | 1.Secondary/High school Diploma  2. Vocational training/College degree 3. A full undergraduate degree (Bachelor, Engineer, Medical Doctor, or equivalent) 4. A full graduate degree (Master and/or PhD, Specialized Professional Degree) 5. Do not know |
|  | **14** | How was your family household (the one you grew up in the first 18 years) classified as one of the followings? | 1. Poor 2. Near poor 3. None of the above |
|  | **15** | Where did you spend most of your years growing up? | List of ward (xã/phường) - district (quận/huyện/thị trấn) - provinces/cities (tỉnh/thành phố) of Vietnam |
|  | **16** | Choose the type of area that you grew up in most of the time during your first 18 years (please choose the most applicable option). | 1. Urban area 2. Rural area |
|  | **17** | How many members are there in your household (where you grew up for the first 18 years)? |  |
|  | **18** | Can you estimate the total average income per person per month of your family household (the one you grew up in the first 18 years)? Please choose a range that you find the most appropriate and representative. | (participants write the number) |

1. Knowledge - **To what extent do you agree or disagree with the following statements:**

| **No.** | **Questions** | **Answers** |
| --- | --- | --- |
| **19** | People with learning disabilities have at least one impaired academic skill of either reading, written expression, or mathematics. | 1. Strongly agree 2. Agree 3. Disagree 4. Strongly disagree |
| **20** | **Some** people with learning disabilities have poorer academic performance compared to what is expected of their age (e.g., having lower test scores, missing deadlines, taking longer time to finish a task or assignment) | 1. Strongly agree 2. Agree 3. Disagree 4. Strongly disagree |
| **21** | **All** people with learning disabilities have poorer academic performance compared to what is expected of their age (e.g., having lower test scores, missing deadlines, taking longer time to complete a task or assignment) | 1. Strongly agree 2. Agree 3. Disagree 4. Strongly disagree |
| **22** | Impairment in academic skills of reading, written expression, and/or mathematics in people with learning disabilities begins during early school years | 1. Strongly agree 2. Agree 3. Disagree 4. Strongly disagree |
| **23** | Symptoms of learning disability tend to become apparent when confronting academic tasks such as timed tests, reading or writing lengthy and complex reports on tight deadlines, or handling excessively heavy academic loads. | 1. Strongly agree 2. Agree 3. Disagree 4. Strongly disagree |
| **24** | People with learning disabilities are also ones who live with intellectual disabilities or some form of mental retardation | 1. Strongly agree 2. Agree 3. Disagree 4. Strongly disagree |
| **25** | Uncorrected visual or hearing problems may cause learning disabilities | 1. Strongly agree 2. Agree 3. Disagree 4. Strongly disagree |
| **26** | Learning disabilities can be successfully treated | 1. Strongly agree 2. Agree 3. Disagree 4. Strongly disagree |
| **27** | An individual with learning disabilities, when provided with appropriate accommodations and support services, may be able to compensate and function better than the ones without. | 1. Strongly agree 2. Agree 3. Disagree 4. Strongly disagree |
| **28** | Based on your understanding, what “learning disability” means to you? |  |

1. Attitude

**In the following scenarios, please respond while regarding the “Learning Disabilities” cases mentioned below are all verified conditions by medical and legal documents.**

**To what extent do you agree or disagree with the following statements:**

| **No.** | **Questions** | **Answers** |
| --- | --- | --- |
| **29** | Providing special considerations during tests to students with verified learning disabilities is unfair to students without learning disabilities. | 1. Strongly agree 2. Agree 3. Disagree 4. Strongly disagree |
| **30** | Providing special considerations during teaching to students with verified learning disabilities is unfair to students without learning disabilities. | 1. Strongly agree 2. Agree 3. Disagree 4. Strongly disagree |
| **31** | Faculties should provide special considerations as necessary for students with learning disabilities who have disclosed their condition. | 1. Strongly agree 2. Agree 3. Disagree 4. Strongly disagree |
| **32** | Faculties should extend the deadlines of assignments to accommodate the needs of students with learning disabilities. | 1. Strongly agree 2. Agree 3. Disagree 4. Strongly disagree |
| **33** | The overall current teaching style in universities permits all students to learn the materials effectively, including students with learning disabilities. | 1. Strongly agree 2. Agree 3. Disagree 4. Strongly disagree |
| **34** | I think it would be appropriate to reduce the overall course reading load for students with learning disabilities. | 1. Strongly agree 2. Agree 3. Disagree 4. Strongly disagree |
| **35** | I think it would be appropriate to let students with learning disabilities complete assignments for “extra credit”. | 1. Strongly agree 2. Agree 3. Disagree 4. Strongly disagree |
| **36** | I think it would be appropriate to grade students with learning disabilities on a different curve. | 1. Strongly agree 2. Agree 3. Disagree 4. Strongly disagree |
| **37** | I think it would be appropriate to allow a student with learning disabilities to substitute an alternative course for a required course. | 1. Strongly agree 2. Agree 3. Disagree 4. Strongly disagree |
| **38** | If a student with learning disabilities did not adequately meet the course requirements despite special considerations, he/she should be given the grade he/she earned. | 1. Strongly agree 2. Agree 3. Disagree 4. Strongly disagree |
| **39** | I think it would be appropriate to allow students with learning disabilities to take proctored exams in a supervised location. | 1. Strongly agree 2. Agree 3. Disagree 4. Strongly disagree |
| **40** | I think it would be appropriate to arrange extended time exams for students with learning disabilities. | 1. Strongly agree 2. Agree 3. Disagree 4. Strongly disagree |
| **41** | I think it would be appropriate to change the method of assessment for students with learning disabilities. | 1. Strongly agree 2. Agree 3. Disagree 4. Strongly disagree |
| **42** | I think it would be appropriate to allow students with learning disabilities to use technology (e.g., laptop, calculator, spell checker) to complete tests even when such technologies are not permitted for use during testing. | 1. Strongly agree 2. Agree 3. Disagree 4. Strongly disagree |
| **43** | I think it would be appropriate to allow students with learning disabilities to tape record. | 1. Strongly agree 2. Agree 3. Disagree 4. Strongly disagree |
| **44** | I believe that students with learning disabilities can complete the university program. | 1. Strongly agree 2. Agree 3. Disagree 4. Strongly disagree |
| **45** | Students with learning disabilities can compete academically with peers at the university level. | 1. Strongly agree 2. Agree 3. Disagree 4. Strongly disagree |
| **46** | I believe that students use learning disabilities as an excuse when they are not doing well in class. | 1. Strongly agree 2. Agree 3. Disagree 4. Strongly disagree |
| **47** | I find that students with learning disabilities wait to talk to **others** until they are not doing well in the class and then I find it hard to believe that they really have a disability. | 1. Strongly agree 2. Agree 3. Disagree 4. Strongly disagree |
| **48** | I find that students with learning disabilities wait to talk to the **faculty** until they are not doing well in the class and then it’s too late for appropriate special considerations to be provided. | 1. Strongly agree 2. Agree 3. Disagree 4. Strongly disagree |

1. Practice

**Please identify if the following practices are being implemented at your university:**

| **No.** | **Questions** | **Answers** |
| --- | --- | --- |
| **49** | My university has an office that provides adequate support for students with disabilities, including those with learning disabilities. | 1. Yes 2. No 3. I don’t know |
| **50** | Teaching faculties in my university invite students to talk to them about learning disability-related issues. | 1. Yes 2. No 3. I don’t know |
| **51** | Teaching faculties in my university spend extra time in class assisting students with learning disabilities. | 1. Yes 2. No 3. I don’t know |
| **52** | Teaching faculties in my university modify their teaching methods to better suit the needs of students with learning disabilities (e.g., providing records of lectures instead of just lecture notes). | 1. Yes 2. No 3. I don’t know |
| **53** | Teaching faculties in my university extend the deadlines of assignments to accommodate the needs of students with learning disabilities. | 1. Yes 2. No 3. I don’t know |
| **54** | Teaching faculties in my university allow students with learning disabilities to take an alternative form of assignments or examination (e.g., oral response instead of essay). | 1. Yes 2. No 3. I don’t know |
| **55** | Teaching faculties in my university allow exams to be proctored in a supervised location other than the classroom for students with learning disabilities. | 1. Yes 2. No 3. I don’t know |
